# *Methanosarcina acetivorans*: A Model for Mechanistic Understanding of Aceticlastic and Reverse Methanogenesis

**DOI:** 10.3389/fmicb.2020.01806

**Published:** 2020-07-28

**Authors:** James G. Ferry

**Affiliations:** Department of Biochemistry and Molecular Biology, Pennsylvania State University, University Park, PA, United States

**Keywords:** global warming, archaea, methane, ecology, evolution, biochemistry, acetate, enzymology

## Abstract

Acetate-utilizing methanogens are responsible for approximately two-thirds of the one billion metric tons of methane produced annually in Earth’s anaerobic environments. *Methanosarcina acetivorans* has emerged as a model organism for the mechanistic understanding of aceticlastic methanogenesis and reverse methanogenesis applicable to understanding the methane and carbon cycles in nature. It has the largest genome in the *Archaea*, supporting a metabolic complexity that enables a remarkable ability for adapting to environmental opportunities and challenges. Biochemical investigations have revealed an aceticlastic pathway capable of fermentative and respiratory energy conservation that explains how *Ms. acetivorans* is able to grow and compete in the environment. The mechanism of respiratory energy conservation also plays a role in overcoming endothermic reactions that are key to reversing methanogenesis.

## Introduction

**FIGURE 1:**
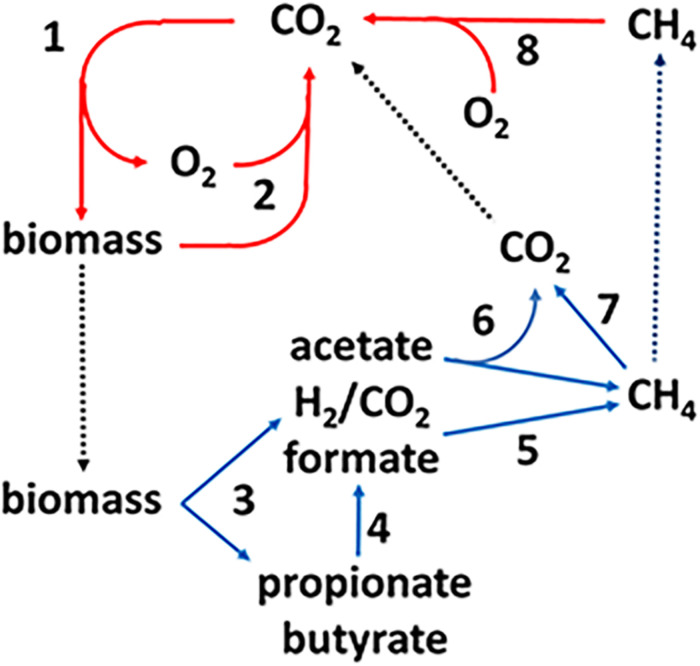
The global carbon cycle. Solid lines indicate aerobic (red) and anaerobic (blue) steps in the cycle and dotted lines indicate transfer of material between aerobic and anaerobic environments. See text for explanation of numbered steps. Not shown are lesser amounts of methane produced from methylotrophic substrates such as methanol and methylamines. Reproduced (Yan and Ferry, 2018).

The production and consumption of CH_4_, the methane cycle, is an important link in the global carbon cycle (Figure 1). The complex biomass produced by photosynthetic plants and microbes is hydrolyzed and oxidized in aerobic habitats by O_2_-respiring microbes producing CO_2_ that re-enters the carbon cycle (steps 1, 2). A fraction of the biomass enters diverse anoxic environments where it is metabolized by microbial food chains comprized of fermentative, acetogenic, and methanogenic anaerobes (steps 3–6) producing an estimated one billion tons of methane (Thauer, 1998). The complex biomass is hydrolyzed and metabolized by fermentative anaerobes that produce primarily acetate plus other higher volatile fatty acids (VFA), H_2_ and formate. The VFA are oxidized to acetate and either formate or H_2_ by acetogens. Thus, acetate is the major metabolite in the food chain that acetotrophic methanogens convert to CH_4_ and CO_2_ (Mah et al., 1977). The balance of global methane production derives primarily from methanogens that oxidize H_2_ or formate and reduce CO_2_ to CH_4_. Methylotrophic methanogens produce minor, although significant, amounts of methane from methyl-containing compounds such as methanol and methylated amines. The CH_4_ produced in anaerobic environments is oxidized to CO_2_ by reversal of methanogenic pathways (step 7). The CO_2_ and residual CH_4_ diffuses into aerobic zones where O_2_ respiring methanotrophs oxidize CH_4_ to CO_2_ thereby closing the carbon cycle (step 8). However, not all the CH_4_ is oxidized and the remaining escapes to the upper atmosphere.

Methane is a greenhouse gas with a global warming potential approximately 20-fold greater than CO_2_ (Ramaswamy et al., 2001). The CH_4_ cycle (production and oxidation) plays an important role in controlling Earth’s climate (Valentine, 2002; Rhee et al., 2009). Indeed, Earths greatest mass extinction is attributed in part to the evolution of acetotrophic methanogens that produced a methanogenic burst in the end-Permian carbon cycle that contributed to a sharp increase in global warming (Rothman et al., 2014). Anthropogenic CH_4_ emissions to the atmosphere have increased sharply since 2007 raising awareness of the potential consequences (Nisbet et al., 2019). A mechanistic biochemical understanding of the CH_4_ cycle is paramount to a deeper understanding necessary to predict and control CH_4_ emissions. Although the understanding of aerobic methanotrophic microbes is well developed, mechanistic understanding of anaerobic CH_4_ oxidation (AOM) is in the early stages.

This review features relevant and recent mechanistic understanding of the aceticlastic pathway and reverse methanogenesis for which *Methanosarcina acetivorans* has emerged as a model.

## Aceticlastic Pathways

**FIGURE 2:**
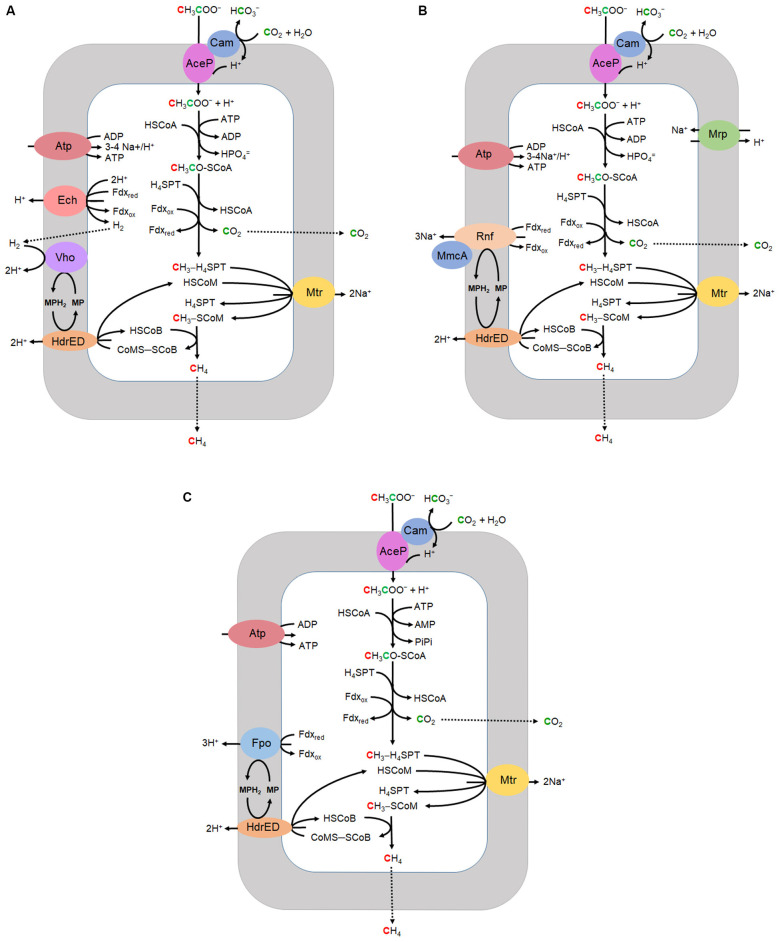
Aceticlastic pathways. **(A)** H_2_ dependent *Methanosarcina*. **(B)** H_2_ independent *Methanosarcina*. **(C)** *Methanothrix*. CoA, coenzyme A; H_4_SPT, tetrahydrosarcinapterin; Fdx, ferredoxin; HSCoM, coenzyme M; HSCoB, coenzyme B; MP, methanophenazine; Cam, gamma carbonic anhydrase; AceP, acetate permease; Mrp, multisubunit sodium/proton antiporter; Atp, ATP synthase; Rnf, homolog of *r*hodobacter *n*itrogen *f*ixation complex; MmcA, multiheme *c*-type cytochrome; HdrED, membrane bound heterodisulfide reductase; Mtr, CH_3_-H_4_SPT:HSCoM methyltransferase; Ech, energy-converting ferredoxin-dependent hydrogenase; Vho, F_420_-nonreactive membrane-bound hydrogenase; Fpo, F_420_H_2_ dehydrogenase multi-subunit complex. Adapted (Smith and Ingram-Smith, 2007).

Most CH_4_ produced in Earth’s diverse anaerobic environments derives from acetate although only two genera, *Methanosarcina* and *Methanothrix* (formerly *Methanosaeta*) are known to grow with acetate and produce CH_4_. Acetotrophic methanogens utilize three variations of the aceticlastic pathway of which two are typical of the genus *Methanosarcina* (*Ms.*) while the third is characteristic of the genus *Methanothrix* (*Mt.*) (Figure 2). All three have in common the transport of acetate, activation to acetyl-CoA, decarbonylation of acetyl-CoA, and one-carbon reactions transforming the methyl group to CH_4_. The variations diverge in the mechanisms of electron transport and energy conservation. Most investigations have centered on *Methanosarcina* for which there are two divergent electron transport pathways, H_2_ dependent and H_2_ independent. The H_2_ dependent pathway (Figure 2A) is well established for *Methanosarcina barkeri* and *Methanosarcina mazei* (Welte and Deppenmeier, 2014). However, the pathway of several acetotrophic *Methanosarcina* species is independent of H_2_ and instead contains the Rnf complex for which *Ms. acetivorans* has emerged as the model (Figure 2B). The Rnf complex is also encoded in all sequenced genomes of diverse methylotrophic genera that includes *Methanosarcina*^1^. Isolated from marine sediment, *Ms. acetivorans* has the largest genome among all methanogens and amenable to robust genetic manipulation (Sowers et al., 1984a; Galagan et al., 2002; Nayak and Metcalf, 2017). The Rnf-dependent aceticlastic pathway of *Ms. acetivorans* (Figure 2B) is supported by transcriptomic, proteomic and modeling investigations (Li et al., 2005a, b; Li et al., 2007; Satish Kumar et al., 2011; Benedict et al., 2012; Peterson et al., 2014).

### Acetate Transport and Activation

AceP from *Ms. acetivorans* was shown to transport acetate by a proton symport mechanism (Ribas et al., 2018). A homolog of AceP was shown to be required for acetate transport of acetate in *Ms. mazei*, and an AceP homolog is encoded in the genome of *Methanothrix thermophila* (Smith and Ingram-Smith, 2007; Welte et al., 2014). The transported acetate is converted to acetyl-CoA by acetate kinase (Ack) and phosphotransacetylase (Pta) in *Methanosarcina*, and by the AMP-forming acetyl-CoA synthetase (Acs) in *Methanothrix* (Berger et al., 2012). It was proposed that Ack and Pta were acquired by horizontal gene transfer from the genus *Clostridium* within the last 475 million years coinciding with evolution of aceticlastic pathways. This event resulted in a significant net increase of CH_4_ leading to climate change in agreement with that proposed for the end-Permian mass extinction (Fournier and Gogarten, 2008; Rothman et al., 2014).

The catalytic mechanism for Ack from *Methanosarcina thermophila* proceeds by nucleophilic attack of the carboxyl group of acetate on the γ-phosphate of ATP with direct in-line transfer to acetate producing acetyl phosphate (Buss et al., 2001; Miles et al., 2002; Ferry, 2011). The mechanism for Pta, also from *Ms. thermophila*, involves base-catalyzed abstraction of the thiol proton of HS-CoA followed by nucleophilic attack of the thiolate anion (^–^S-CoA) on the carbonyl carbon of acetyl phosphate forming acetyl-CoA (Iyer et al., 2004; Lawrence et al., 2006; Ferry, 2011). The crystal structure and biochemical characterization of Acs from *Ms. acetivorans* revealed the preference for medium chain substrates that excludes acetate, a result which indicates Acs functions other than activating acetate to acetyl-CoA (Ingram-Smith and Smith, 2007; Shah et al., 2009; Meng et al., 2010). The Acs of *Methanothrix* has a greater affinity for acetate than Ack of *Ms. acetivorans* which explains the dominance of *Methanothrix* in environments where acetate is in concentrations <0.1 mM (Berger et al., 2012). The acetyl-CoA is decarbonylated by the acetyl-CoA decarbonylase/synthase (ACDS) yielding a methyl group and CO. The methyl group is transferred to tetrahydrosarcinapterin (H_4_SPT) yielding CH_3_-H_4_SPT and CO is oxidized to CO_2_ with transfer of electrons to either ferredoxin (Fdx) or a novel flavodoxin (FldA) characterized from *Ms. acetivorans* (Prakash et al., 2019b).

The ACDS is predicted to be a component of the last universal common ancestor (LUCA) (Adam et al., 2018). Although of ancient origin and of central importance in the aceticlastic pathway, an atomic resolution structure of the intact ACDS complex from any methanogen is not reported. The enzymes from *Methanosarcina* and *Methanothrix* are known to have five subunits (αβγδε) based on the purified complexes and genomic analyses (Terlesky et al., 1986; Grahame and Demoll, 1996; Smith and Ingram-Smith, 2007). The β subunit catalyzes decarbonylation of acetyl-CoA while the αε subunits catalyze CO oxidation and the γδ subunits transfer the methyl group to H_4_SPT producing CH_3_-H_4_SPT (Murakami and Ragsdale, 2000). The crystal structure of the αε component of *Ms. barkeri* identified the active site in the α subunit comprised of a pseudocubane Ni-Fe_3_S_4_ cluster bridged to an exogenous iron atom (Gong et al., 2008). A mechanism was proposed wherein the CO bound to Ni, and the OH^–^ bound to exogenous iron, H are coupled to form CO_2_. A role for the ε subunit was proposed in which bound FAD directs electrons from the α subunit to Fdx. This proposal fits with the possibility that FldA accepts electrons from the ε subunit of the ACDS from *Ms. acetivorans* at the proposed FAD site. Spectroscopic studies of the β subunit from *Ms. thermophila* indicate an active site Fe_4_S_4_ cluster bridged to a binuclear Ni–Ni site in analogy to the homolog from an acetogen of the domain *Bacteria* that synthesizes acetyl-CoA (Gu et al., 2003; Funk et al., 2004; Ragsdale, 2007). Kinetic and EPR spectroscopy results indicate that alterations in the Ni coordination environment of the active site cluster promote C–C bond cleavage dependent on conformational changes (Gencic and Grahame, 2008). The γδ component transfers the methyl group of acetyl-CoA to H_4_SPT involving a corrinoid coenzyme, although it is unknown which subunit interacts with H_4_SPT and a crystal structure is not available (Grahame, 1993).

Acetate-grown *Ms. acetivorans* up regulates a γ class carbonic anhydrase (Cam) for which the crystal structure and biochemical characterization of the homolog from *Ms. thermophila* revealed the catalytic mechanism involving an active-site iron (Kisker et al., 1996; Iverson et al., 2000; Macauley et al., 2009; Zimmerman et al., 2013). Although homologs are present in acetate grown *Methanosarcina* and *Methanothrix*, the physiological function is not established. A plausible function involves diffusion of cytoplasmic CO_2_ to the outer aspect of the membrane where AceP is located in a complex with Cam that hydrates CO_2_ to ${\text{HCO}}_{3}^{-}$/H^+^ which supplies a local concentration of protons for symport of acetate by AceP (Figure 2). In this way, the proton gradient that drives ATP synthesis is not collapsed. The putative function for Cam is analogous to that reported for the α class carbonic anhydrase that supplies a proton for symport of lactate in mammalian cells (Peetz et al., 2014).

### One-Carbon Reactions

The methyl group of CH_3_-H_4_SPT is transferred to coenzyme M (HS-CoM) coupled to sodium extrusion by a membrane bound methyltransferase (MtrABCDEFGH). The CH_3_-SCoM is reductively demethylated to CH_4_ by the methyl coenzyme M reductase (McrABG) requiring coenzyme B (HSCoB) as the reductant. Post-translational modified residues *N^1^-*methylhistidine (3-methylhistidine), 5-(S)-methylarginine, thioglycine, and *S*-methylcysteine are present in the active-sites of the catalytic McrA subunits from phylogenetically and metabolically diverse methanogenic and methanotrophic archaea (Grabarse et al., 2000; Kahnt et al., 2007). Mcr from *Ms. acetivorans* has emerged as a model for investigations of the modified residues. A unique radical SAM methyltransferase was shown required for methylation of the active-site arginine and concluded important for stability under imposed oxidative and heat stress (Deobald et al., 2018; Radle et al., 2019). Deletion of a homolog essential for arginine methylation in the obligate CO_2_-reducing methanogen *Methanococcus maripaludis* resulted in a 40–60% loss in the rate of methanogenesis consistent with partial loss of Mcr activity (Lyu et al., 2020). Deletion of two genes essential for thioglycine synthesis in McrA of *Ms. acetivorans* produced mutants severely impaired in the rate of growth with acetate and when exposed to thermal and oxidative stress, results supporting a role for thioglycine in stabilizing the McrA active-site although not essential. Combinatorial deletion of genes responsible for incorporation of 5-(S)-methylarginine, thioglycine and *S*-methylcysteine generated *Ms. acetivorans* mutants with phenotypes consistent with altered thermal stability of McrA (Nayak et al., 2020). The studies suggest that residue modifications of Mcr function in important ways although not essential for catalysis. The CoMS-SCoB product of Mcr is reduced by a membrane bound electron transport chain ending with heterodisulfide reductase (HdrE_1_D_1_) that regenerates sulfhydryl forms of the coenzymes.

### Electron Transport and Energy Conservation

The electron transport pathways of all acetotrophic methanogens begin with the oxidation of Fdx and end with reduction of CoMS-SCoB by HdrE_1_D_1_ (Figure 2). As heterodisulfide is the terminal electron acceptor and generated internally, the process fits the definition of fermentative electron transport and energy conservation as opposed to respiration that requires an externally supplied electron acceptor. The aceticlastic pathways diverge in the mechanisms of membrane-bound electron transport that generates ion gradients driving ATP synthesis for growth (Figure 2). The H_2_ dependent pathway (Figure 2A) has been investigated in *Ms. barkeri* and *Ms. mazei* for which the understanding is well developed (Welte and Deppenmeier, 2014). Reduced Fdx donates electrons to Ech hydrogenase that pumps protons and also reduces protons to H_2_ that diffuses across the membrane where it is reoxidized at the outer aspect by the Vho hydrogenase, further contributing to the proton gradient (Welte and Deppenmeier, 2014; Kulkarni et al., 2018). Electrons from the oxidation of H_2_ by Vho are transferred to HdrE_1_D_1_ by the quinone-like electron carrier methanophenazine (MP) accompanied by the vectoral translocation of protons that supplements the proton gradient. The proton gradient, together with the Mtr imposed Na^+^ gradient, drives ATP synthesis.

**FIGURE 3:**
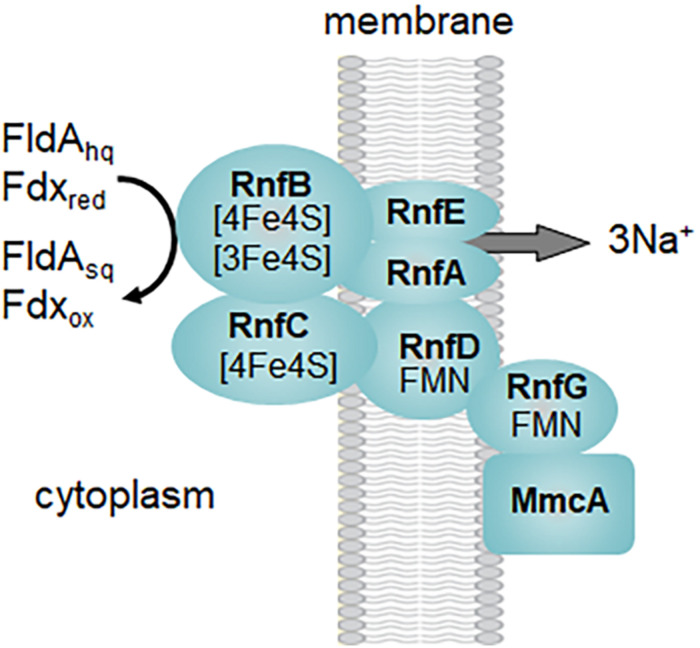
Proposed organization of the Rnf complex from *Methanosarcina acetivorans*. Symbols: FldA_sq_, semiquinone of flavodoxin A; FldA_hq_, hydroquinone of flavodoxin A; Fdx_ox_, oxidized ferredoxin; Fdx_red_, reduced ferredoxin. Modified (Suharti et al., 2014).

Several acetotrophic *Methanosarcina* lack Ech and Vho hydrogenases and are H_2_ independent (Zhilina, 1978; Ollivier et al., 1984; Sowers et al., 1984a; Zinder et al., 1985; Elberson and Sowers, 1997; Von Klein et al., 2002; Shimizu et al., 2011; Ganzert et al., 2014). *Ms. acetivorans* is typical of H_2_ independent *Methanosarcina* that instead utilize the membrane bound RnfCDGEAB complex to oxidize Fdx or FldA (Figure 2B; Li et al., 2006; Wang et al., 2011; Schlegel et al., 2012b; Prakash et al., 2019b). FldA accepts electrons from ACDS and is proposed to replace Fdx when growing in iron-limited environments (Prakash et al., 2019b). Fdx is an electron donor to the RnfB subunit of the Rnf complex (Suharti et al., 2014). It was further shown that the heterologously produced flavin-containing RnfG subunit is located on the outer aspect of the *Escherichia coli* membrane leading to the proposed model shown in Figure 3. Although MmcA is abundant in acetate-grown cells, its role in acetotrophic growth is questioned with the finding that a Δ*mmcA* mutant grows with acetate (Holmes et al., 2019). In contrast, the mutant is incapable of methanol-dependent respiratory growth with anthraquinone-2,6-disulfonate (AQDS), which suggests a role for MmcA in mediating electron transfer to external electron acceptors which fits the definition of respiratory electron transport and energy conservation. Rnf transfers electrons to MP for reduction of CoMS-SCoB by HdrE_1_D_1_ and pumps Na^+^ that thermodynamic considerations predict 3-4Na^+^/2 electrons (Schlegel et al., 2012b; Welte and Deppenmeier, 2014). Thus, electron transport generates H^+^ and Na^+^ gradients that, together with the Mtr-imposed Na^+^ gradient, drives ATP synthesis by the ATP synthase dependent on both H^+^ and Na^+^ (Schlegel et al., 2012a). It is proposed that the multi subunit Na^+^/H^+^ antiporter MrpABCDEFG adjusts the Na^+^/H^+^ ratio optimal for ATP synthesis (Jasso-Chavez et al., 2013, 2017). Although electron transport is remarkably different in *Ms. barkeri* and *Ms. acetivorans*, they have similar growth rates and yields in the absence of an exogenous electron acceptor which indicates that each conserve the same amount of energy (Sowers et al., 1984b). This result is consistent with equivalent H^+^ and Na^+^ gradients generated by electron transport and Mtr.

*Methanosarcina acetivorans, Ms. barkeri* and *Ms. mazei* each encode HdrE_1_D_1_, HdrA_1_B_1_C_1_, HdrD_2_, HdrA_2_, and HdrC_2_B_2_. HdrE_1_D_1_ was shown to function in acetotrophic growth of *Ms. acetivorans* whereas HdrA_1_B_1_C_1_ is apparently specific for methylotrophic growth (Buan and Metcalf, 2010; Catlett et al., 2015). It is proposed that reduced Fdx, generated in the oxidative branch, donates electrons to HdrA_1_B_1_C_1_ that then reduces F_420_ at the expense of CoMS-SCoB reduction in an electron bifurcation reaction (Buan and Metcalf, 2010). With this mechanism, electrons from Fdx are directed to the Fpo complex which results in additional energy conservation. A mechanism is proposed for the catalytic subunit HdrD that is distinct from the catalytic HdrB of the electron bifurcating HdrABC of obligate CO_2_-reducing methanogens. Based on the crystal structure alone, a mechanism is proposed for HdrB involving two novel non-cubane 4Fe4S clusters (Wagner et al., 2017). This mechanism contrasts with that proposed for HdrD involving one conventional 4Fe4S cluster although based primarily on spectroscopic analyses (Walters and Johnson, 2004). However, both mechanisms propose that on reduction of CoMS-SCoB the sulfur atoms of the HSCoM and HSCoB are bound to iron in a five-coordinate manner. The electron pair for reduction of CoMS-SCoB derives from a membrane-bound electron transport chain that accepts electrons from either reduced Fdx or a flavodoxin (FldA) generated by ACDS (Figure 2B). The HdrE_1_ subunit contains a *b*-type cytochrome that accepts electrons from MP for transfer to HdrD_1_ (Welte and Deppenmeier, 2014).

**FIGURE 4:**
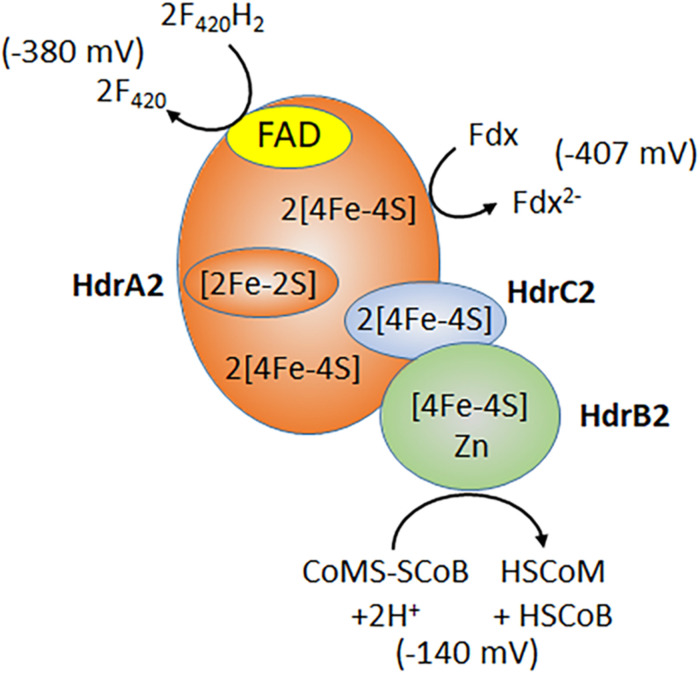
Electron bifurcation by HdrA_2_B_2_C_2_. F_420_, coenzyme F_420_; Fdx, ferredoxin; HSCoM, coenzyme M; HSCoB, coenzyme B. Redox potentials for F_420_ and CoMS-SCoB are published values (Thauer et al., 2008). The ferredoxin redox potential is determined for the 2(4Fe4S) ferredoxin from acetate grown *Ms. thermophila* (Clements et al., 1994). Modified (Yan et al., 2017).

Subunits of the recently characterized electron bifurcating HdrA_2_B_2_C_2_ are up regulated in acetate-grown *Ms. acetivorans* consistent with a role in acetotrophic growth (Li et al., 2007; Buan and Metcalf, 2010; Rohlin and Gunsalus, 2010; Yan et al., 2017). Indeed, acetotrophic growth is impaired in a strain of *Ms. acetivorans* unable to synthesize HdrA_2_B_2_C_2_ (Buan and Metcalf, 2010). Expression of the individual HdrA_2_, HdrB_2_, and HdrB_2_C_2_ subunits in *E. coli*, and biochemical characterization of the reconstituted active HdrA_2_B_2_C_2_ complex, revealed a role for HdrA_2_ in the oxidation of reduced coenzyme F_420_ (F_420_H_2_) and FAD-dependent bifurcation of electrons that are transferred to Fdx and HdrC_2_ (Figure 4; Yan et al., 2017). The HdrC_2_ mediates electron transfer to HdrB_2_ for reduction of CoMS-SCoB. The thermodynamically unfavorable reduction of Fdx is driven by the more favorable reduction of CoMS-SCoB. Although up regulated in acetate grown cells, the role for HdrA_2_B_2_C_2_ in acetotrophic growth has not been established experimentally. It is postulated that the Rnf complex reduces coenzyme F_420_ that is oxidized by HdrA_2_B_2_C_2_ thereby recycling electrons to Fdx for oxidation by Rnf and an additional Na^+^ translocated, improving the thermodynamic efficiency (Buckel and Thauer, 2018). An unusual flavodoxin (FldA) can replace Fdx as electron donor to Rnf and acceptor for HdrA_2_B_2_C_2_ (Prakash et al., 2019b). FldA is a potential advantage in periods of oxidative stress that damage the iron-sulfur clusters of Fdx, or when iron is limiting in the environment (Prakash et al., 2019b).

Considerably less is known of electron transport and energy conservation in *Methanothrix*. The genomes are void of genes encoding Ech hydrogenase or Rnf and, instead, encode F_420_H_2_ dehydrogenase (FpoABCDHIJKLMNO) although lacking the gene encoding FpoF that in *Methanosarcina* is the input module oxidizing F_420_H_2_ (Zhu et al., 2012). Thus, it is postulated that Fpo accepts electrons directly from Fdx with MP-mediated reduction of HdrED that is encoded in *Methanothrix* genomes (Zhu et al., 2012). Thermodynamic considerations predict 3H^+^ translocated by Fpo for a total of seven ions contributing to the gradient driving ATP synthesis (Welte and Deppenmeier, 2014). Although equivalent to gradients generated by H_2_ dependent and H_2_ independent *Methanosarcina* (Figure 2), *Methanothrix* requires two ATP for activation of acetate compared to one for *Methanosarcina* which predicts lower growth yields. However, this thermodynamic disadvantage is at least partially compensated by the ability of *Methanothrix* to metabolize acetate at lower concentrations compared to *Methanosarcina* (Jetten et al., 1992).

### Respiratory Energy Conservation

**FIGURE 5:**
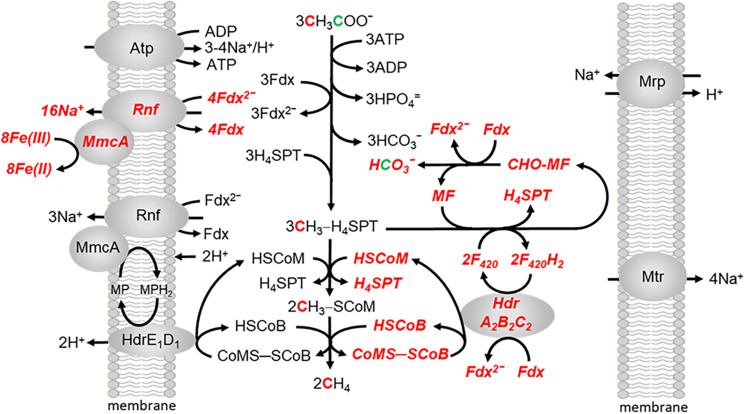
The aceticlastic pathway proposed for growth of *Ms. acetivorans* in the presence of ferrihydrite. Respiratory electron transport is shown in bolded italicized red font. Modified (Prakash et al., 2019a).

*Methanosarcina acetivorans* is capable of Fe(III)-dependent respiratory growth with acetate, a finding previously undocumented for acetotrophic methanogens (Prakash et al., 2019a). Growth and acetate consumption nearly doubles in the presence of ferrihydrite [Fe(OH)_3_], the metal oxide form of Fe(III) that is common in the environment. Ferric iron is stoichiometrically reduced to ferrous iron. The ATP/ADP ratio also doubles indicating a higher energetic state consistent with increased growth. However, CH_4_ is also produced indicating both fermentative and respiratory electron transport and energy conservation. The revised, ecologically relevant, pathway is shown in Figure 5. All one-carbon transformations leading to CH_4_ are the same as in Figure 2. Two Na^+^ are translocated for each Fe(III) reduced to Fe(II) in respiratory electron transport (Yan et al., 2018). Although further research is necessary, the present results indicate that productive Na^+^ translocation by the Rnf complex is dependent on electron transfer to MmcA that reduces an exogenous electron acceptor which fits the definition of respiratory electron transport. Respiratory electron transport is dependent on oxidation of the methyl group from CH_3_-H_4_SPT by reversal of reactions in the CO-dependent pathway of CO_2_ reduction to CH_4_ and acetate in *Ms. acetivorans* which generates reduced coenzyme F_420_ (F_420_H_2_) and additional reduced Fdx to enter the pool for both respiratory and fermentative electron transport (Lessner et al., 2006). The F_420_H_2_ dehydrogenase, essential for methylotrophic growth, is down regulated in acetate-grown cells leading to the proposal that oxidation of F_420_H_2_ is dependent on the electron bifurcating HdrA_2_B_2_C_2_ (Yan et al., 2017). As FldA can replace Fdx as electron acceptor for HdrA_2_B_2_C_2_, and donor to Rnf, either are available for initiating fermentative and respiratory electron transport (Prakash et al., 2019b). The combination of fermentative and respiratory electron transport generates both H^+^ and Na^+^ gradients that drive ATP synthesis by the ATP synthase dependent on both gradients (Schlegel et al., 2012a). It is proposed that the multi subunit Na^+^/H^+^ antiporter Mrp adjusts the Na^+^/H^+^ ratio optimal for ATP synthesis (Jasso-Chavez et al., 2013, 2017).

**FIGURE 6:**
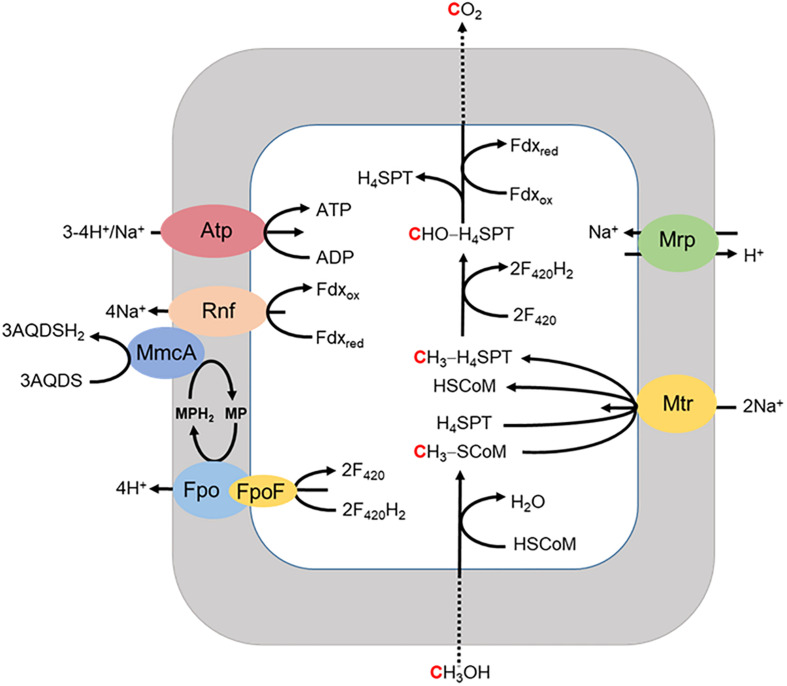
Proposed model for extracellular electron transport to AQDS by *Ms. acetivorans* grown with methanol in the presence of the methanogenesis inhibitor 2-bromoethanesulfonic acid (BES). FpoF, input module to Fpo. Adapted (Holmes et al., 2019).

A respiratory pathway is also proposed for *Ms. acetivorans* grown with methanol when methanogenesis is inhibited by 2-bromoethanesulfonate (Figure 6; Holmes et al., 2019). The methyl group of methanol is oxidized to CO_2_ with reduction of Fdx and F_420_ for which the latter is reoxidized by the F_420_H_2_ dehydrogenase complex (Fpo and FpoF) that is up regulated in methanol grown cells. Fpo transfers the electrons to MP accompanied by the translocation of H^+^ which contributes to the ion gradient that drives ATP synthesis. Reduced MP transfers electrons to MmcA that reduces AQDS as the final electron acceptor. The reduced Fdx donates electrons to Rnf that also transfers electrons to MmcA with translocation of Na^+^ analogous to that proposed in the revised aceticlastic pathway (Figure 4). The imposed inhibition of methanogenesis precludes extrapolation to the environment although reinforces the discovery that *Ms. acetivorans* is capable of respiratory growth.

### Ecology and Evolution

The revised aceticlastic pathway of *Ms. acetivorans* has important ecological and evolutionary implications. Without respiration, the amount of energy

(1) $${\text{CH}}_{3}{\text{ CO}}_{2}\text{ H}\to {\text{CO}}_{2}+{\text{CH}}_{4}\left(\Delta {\text{G}}^{\circ ,\prime }=-36\text{kJ/mol}\right)$$

(2) $$\text{ADP}+\text{Pi}\to \text{ATP}+{\text{H}}_{2}\text{O}\left(\Delta {\text{G}}^{\circ ,\prime }=+31.8\text{kJ/mol}\right)$$

available by methanogenesis alone, with equimolar reactants and products (Eq. 1), is barely enough to synthesize one ATP (Eq. 2). It is possible that growth by methanogenesis alone is only achievable in the laboratory with an abundant supply of acetate at optimal temperature, pH, and supply of nutrients whereas growth in the competitive and dynamic environment is dependent on additional energy gained by respiration. In environments where Fe(III) is limiting, energy conservation by methanogenic fermentation could afford an advantage over acetotrophic competitors that conserve energy only by respiration. *Ms. acetivorans*, and other *Methanosarcina* which are H_2_ independent, may have an advantage over H_2_ dependent *Methanosarcina* that are without multi-heme c-type cytochromes and incapable of respiratory growth.

## Reverse Methanogenesis

**FIGURE 7:**
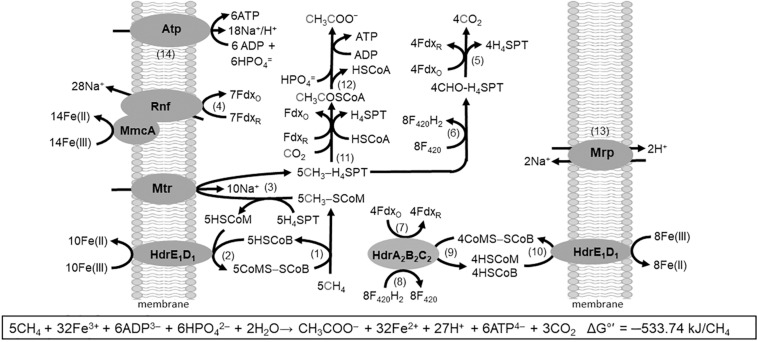
Pathway proposed for Fe(III)-dependent CH_4_ oxidation, electron transport, and conservation of energy by *Ms. acetivorans*. See text for explanation of numbered steps. Not shown is AQDS-mediated reduction of Fe(III) at MmcA and HdrE_1_D_1_. Modified (Yan et al., 2018).

The discovery of respiratory energy conservation by *Ms. acetivorans* has impacted understanding of reverse methanogenesis, the CH_4_ cycle, and the iron cycle in nature. Previous models of the anaerobic oxidation of CH_4_ (AOM) involved anaerobic methanotrophic archaea (ANME) that oxidize CH_4_ by reversal of the CO_2_-reduction pathway of methanogens. The oxidation required a symbiosis with species utilizing reductant produced by ANME to make the overall reaction thermodynamically favorable. However, it was found that AQDS decouples CH_4_ oxidation from sulfate reduction which presented the possibility of independent respiratory methanotrophic growth by ANME. *Ms. acetivorans* is capable of trace CH_4_ oxidation during growth with methanogenic substrates (Moran et al., 2005, 2007). Furthermore, *Ms. acetivorans* is capable of Fe(III)-dependent AOM in the absence of methanogenic substrates when engineered with the Mcr gene derived from ANME-1 sediment (Soo et al., 2016). Biochemical investigations support a proposed AOM pathway for *Ms. acetivorans* anchored by Fe(III)-dependent mechanisms for energy conservation that drive endergonic reactions essential for methanotrophic growth (Figure 7) (Yan et al., 2018).

The AOM pathway postulates that CH_4_ is oxidized by Mcr producing CH_3_-SCoM (Rxn. 1) in analogy to that shown for the Mcr of obligate CO_2_-reducing methanogens (Scheller et al., 2010). The exergonic Fe(III)-dependent oxidation of HSCoM and HSCoB by HdrE_1_D_1_ (Rxn. 2) drives the endergonic oxidation of CH_4_ (Yan et al., 2018). The endergonic methyl transfer from CH_3_-SCoM to H_4_MPT by Mtr (Rxn. 3) is driven with the Na^+^ gradient generated by the Rnf complex (Rxn. 4) with a stoichiometry of 2Na^+^ translocated per electron transferred from Fdx to Fe(III) (Yan et al., 2018). Electrons are transferred from Rnf to MmcA that reduces Fe(III). Reduced Fdx is a product of the oxidation of the methyl group of CH_3_-H_4_SPT to CO_2_ (Rxn. 5) as is also F_420_H_2_ (Rxn. 6) that is oxidized by HdrA_2_B_2_C_2_ (Rxn. 7) with reduction of Fdx (Rxn. 8) and CoMS-SCoB (Rxn. 9). The CoMS-SCoB is regenerated (Rxn. 10) as for the Fe(III)-dependent oxidation of HSCoM and HSCoB by HdrE_1_D_1_ (Rxn. 2). Reactions oxidizing the methyl group of CH_3_-H_4_MPT to CO_2_ (Rxn. 5 and 6) are the reverse of reactions in the CO-dependent pathway of CO_2_ reduction to CH_4_ and acetate in *Ms. acetivorans* (Lessner et al., 2006). Reactions leading from CH_3_-H_4_MPT to acetate (Rxn. 11 and 12) are the reverse of reactions in the aceticlastic pathways (Figures 2, 4). The Na^+^/H^+^ antiporter Mrp is postulated to adjust the Na^+^/H^+^ ratio optimal for ATP synthesis by the Atp synthase dependent on both Na^+^ and H^+^ gradients (Rxn. 13 and 14) (Schlegel et al., 2012a; Jasso-Chavez et al., 2013, 2017). Not shown in Figure 7 is the requirement for AQDS to mediate electron transfer from HdrE_1_D_1_ to Fe(III) and MmcA to Fe(III). AQDS is an analog of humic substances that are proposed to replace AQDS in nature (Holmes et al., 2019).

The pathway resembles the AOM pathway predicted for an uncultured ANME-2a based on metagenomic analyses (Wang et al., 2014). However, it should be cautioned that the biochemistry of ANME is largely unknown and differences with methanogenic pathways are anticipated (Timmers et al., 2017). Nonetheless, the biochemical-based AOM pathway provides a working model for mechanistic understanding of the growing literature describing respiratory AOM by individual ANME using a variety of electron acceptors including Fe(III) (Raghoebarsing et al., 2006; Beal et al., 2009; Haroon et al., 2013; Ettwig et al., 2016; Cai et al., 2018; He et al., 2018; Liang et al., 2019; Luo et al., 2019; Aromokeye et al., 2020; Leu et al., 2020).

### Ecology and Evolution

The realization of Fe(III)-dependent AOM has implications for understanding the CH_4_ and iron cycles, both past and present. It is postulated that symbiotic associations of ANME and sulfate-reducing species evolved from methanogenic species that first acquired the capacity to conserve energy by oxidizing CH_4_ and reducing metals (Scheller et al., 2016). Moreover, it is postulated that Fe(III)-dependent AOM was largely responsible for oxidizing all the CH_4_ produced on early Earth prior to the appearance of oxygen (Beal et al., 2009). It is further hypothesized that if only a small fraction of current global Mn(IV) and Fe(III) influx is used for AOM, it has the potential to consume a large amount of CH_4_ (Beal et al., 2009). *Ms. acetivorans* was isolated from off shore marine sediments near locations with CH_4_ seeps where single cells and aggregates of ANME are present and could play a role in non-symbiotic Fe(III)-dependent AOM (Sowers et al., 1984a; Orphan et al., 2002).

## Conclusion

Acetotrophic methanogens utilize three aceticlastic pathways separated by mechanisms of electron transport and energy conservation that are well developed for the genus *Methanosarcina* and less so for *Methanothrix*. *Ms. acetivorans* is a model for H_2_ independent mechanisms whereas *Ms. mazei* and *Ms. barkeri* are models for the H_2_ dependent mechanisms. Recent developments establish respiratory energy conservation for *Ms. acetivorans* dependent on a multi-heme *c*-type cytochrome explaining growth in the environment and further separating H_2_ independent and H_2_ dependent *Methanosarcina*. However, gaps remain in our understanding of aceticlastic catabolism in *Methanosarcina* which include the mechanism of HdrED, a complete structure and mechanism for ACDS, and electron transport from multi-heme *c*-type cytochrome to exogenous electron acceptors.

## Author Contributions

JF wrote the review.

## Conflict of Interest

The author declares that the research was conducted in the absence of any commercial or financial relationships that could be construed as a potential conflict of interest.

## Footnotes

1 https://pubmed.ncbi.nlm.nih.gov/
